# The association between dietary fiber intake and severe headaches or migraine in US adults

**DOI:** 10.3389/fnut.2022.1044066

**Published:** 2023-01-04

**Authors:** Hao Huang, Kaiyin He

**Affiliations:** ^1^Department of Pain Management, The First Affiliated Hospital, Jinan University, Guangzhou, Guangdong, China; ^2^Department of Clinical Nutrition, The First Affiliated Hospital, Jinan University, Guangzhou, Guangdong, China

**Keywords:** dietary fiber, migraine, headaches, NHANES, race

## Abstract

**Background:**

The data on the effect of dietary fiber on severe headaches or migraine are limited. Therefore, this study aimed to investigate the association between dietary fiber intake and the prevalence of severe headaches or migraine.

**Methods:**

We conducted a cross-sectional study involving 12,710 participants, all data collected from NHANES 1999–2004. A multivariable logistic regression model was used to analyze the relationship between dietary fiber intake (as an independent variable) and severe headaches or migraine (as outcome variable). We also performed sensitivity analyses, including multiple sensitivity analyses.

**Results:**

The overall incidence of severe headache or migraine in the study was 2527/12,710 (19.9%). After adjusting for correlation covariates, we found a significant inverse association between dietary fiber intake and severe headache or migraine, with lowest prevalence in the fifth quintile (OR: 0.74, 95% CI: 0.61–0.90). Our study also revealed that for every 10 g/day increase in dietary fiber intake, the prevalence of severe headache or migraine decreased by 11%. However, no such inverse association was found among Mexican Americans, other races, or those with a body mass index (BMI) of 25–30. *E*-value analysis suggested robustness to unmeasured confounding.

**Conclusion:**

Increasing the intake of fiber-rich foods might protect from severe headache or migraine. More prospective studies should be conducted to confirm their association before dietary recommendations.

## Introduction

Migraine is a highly disabling primary headache disease, which causes more disabilities than all other neurological diseases combined (1). The global prevalence rate is estimated to be 15–18%, causing a high burden on individuals, families, health care systems, and societies (2). Genetic and environmental factors play an important role in the occurrence of migraine (3). In addition, a variety of other internal and external conditions, such as dietary patterns, specific foods, alcohol, stress and other lifestyle factors, can influence the occurrence, intensity and duration of migraines (4, 5). In recent years, the Global Burden of Disease (GBD) consortium has concluded that sub-optimal diets are responsible for more deaths from non-communicable diseases worldwide than any other risk factor, including smoking. Thus, promoting the consumption of dietary components with below-optimal intakes is considered an effective way to reduce the burden of disease associated with dietary risk (6). Statistically, dietary fiber intake is indeed consistently reported as inadequate compared to recommended intakes, regardless of country (7).

Dietary fiber is composed of carbohydrate polymers, which can neither be digested nor absorbed in human intestines, but enters the large intestine and is fermented by intestinal microbiota (8). Because dietary fiber is complex and heterogeneous, it can be divided into four subgroups according to different chemical structures, physicochemical characteristics, and degree of polymerization: resistant oligosaccharides, non-starch polysaccharides, resistant starch, and associated substances (non-carbohydrates) (7). Foods rich in dietary fibers include fruits, vegetables, whole grains, potatoes, tubers, and beans. Although dietary fiber has different subgroups, there is no disagreement in the consensus that it is beneficial to human health. The health benefits of dietary fiber include improving intestinal function, reducing blood glucose concentration, lowering blood cholesterol and increasing satiety. There is increasing evidence that dietary fiber plays a role in reducing mortality and chronic diseases such as cardiovascular disease and type 2 diabetes mellitus (9).

In addition, dietary fiber has the ability to regulate the intestinal microbiome. Recent research suggests a bidirectional relationship between gut microbiota and the brain. Disturbances in the gut microbiota are closely linked to central nervous system diseases, such as multiple sclerosis, Parkinson’s disease, Alzheimer’s disease, depression etc. (10).

According to neuroimaging research, migraine is a multifaceted, central nervous system disorder (11). However, to the best of our knowledge, there are still no studies investigating the relationship between dietary fiber intake and migraine. The aim of this study was to determine the relationship between dietary fiber intake and migraine after adjustment for potential confounders. In addition, the study further examined the stability of the relationship between dietary fiber intake and migraine by gender, age, and race.

## Materials and methods

### Study design and participants

This is a cross-sectional study with data from the National Health and Nutrition Examination Survey (NHANES) 1999–2004. NHANES is a population-based, nationwide cross-sectional survey conducted annually by the National Center for Health Statistics of the Centers for Disease Control and Prevention and published every 2 years. The NHANES program has been approved by the Ethics Review Board of the National Center for Health Statistics research, and participants are required to sign an informed consent form. All NHANES data and more details can be found on the NHANES website.^1^

**FIGURE 1:**
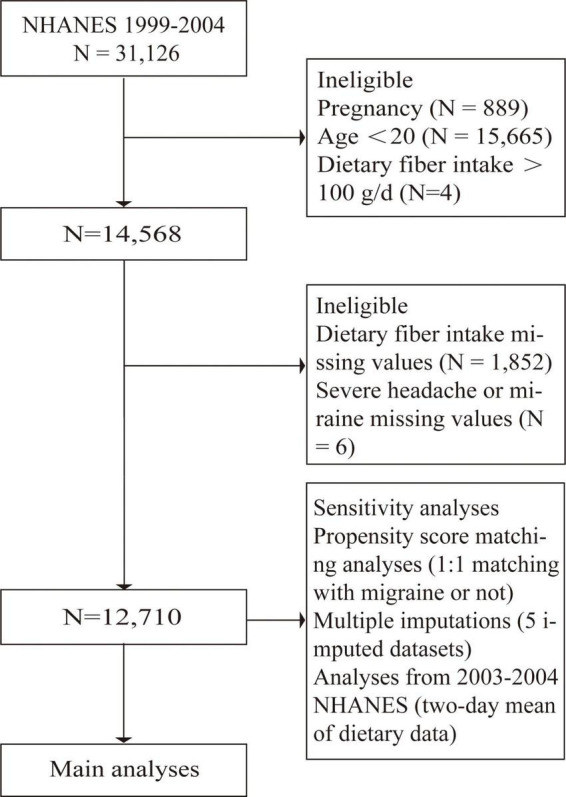
Flow chart (raw data).

The 1999–2004 NHANES participants were included in our study. Exclusion criteria were <20 years of age, pregnancy, fiber intake of more than 100 g/day, and missing information about migraine (or severe headache) or fiber intake (Figure 1).

### Assessment of headaches or migraines

Severe headaches or migraines as dependent variables were assessed in the miscellaneous pain section of the NHANES questionnaire on a self-reported basis. For the question “have you ever had a severe headache or migraine in the past 3 months?,” Participants who answered “yes” were considered to have severe headaches and migraines.

### Assessment of dietary intakes

National Health and Nutrition Examination Survey assessed participants’ dietary intake (including types and quantities of food) by a 24-h dietary recall, conducted by trained interviewers. Dietary recall interviews were conducted in a Mobile Examination Center with a standardized set of guidelines. In 1999–2002, the NHANES conducted one 24-h recall as part of the interview, while in 2003–2004, two 24-h recalls were recorded (the second dietary interview over the phone was conducted 3–10 days later). To be consistent with data from 1999 to 2002, we use only data from the first interview in 2003–2004. Therefore, we performed the main analyses based on the NHANES 1999–2004 first-day dietary data. In addition, as two 24-h dietary recalls were conducted in 2003–2004, we further performed sensitivity analyses of the mean values of these two dietary intakes (Supplementary Table 1). NHANES calculated each subject’s total energy and intake of nutrients using the U.S. Department of Agriculture Food and Nutrient Database.

### Covariates

The following variables were included in the analysis as covariates: age (smooth), body mass index (BMI) (smooth), gender, race, education levels, C reactive protein (smooth), total energy intake (smooth), family poverty income ratio (smooth), alcohol intake (smooth), protein intake (smooth), carbohydrate intake (smooth), history of hypertension, cancer or malignancy, coronary heart disease, stroke, physical activity, smoked at least 100 cigarettes in life.

Multiple imputation (MI) is an effective approach to deal with missing covariable data. In our sensitivity analyses, MI were conducted using R multivariate imputation by chain equation (MICE) package (Supplementary Tables 2, 3). Missing data patterns, summaries of raw and imputed data, and visualization of non-missing data for all covariates in the raw data are shown in Supplementary Tables 3–5 and Supplementary Figure 1. Variables description and coding were shown in Supplementary Table 6.

### Statistical analysis

**FIGURE 2:**
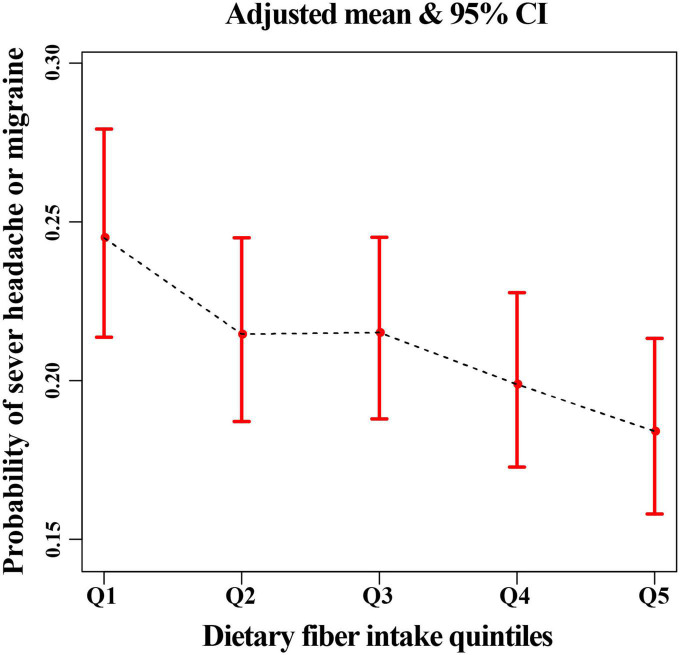
The relationship between headache or migraine with dietary fiber quintiles after adjusting all related covariates in this study.

**FIGURE 3:**
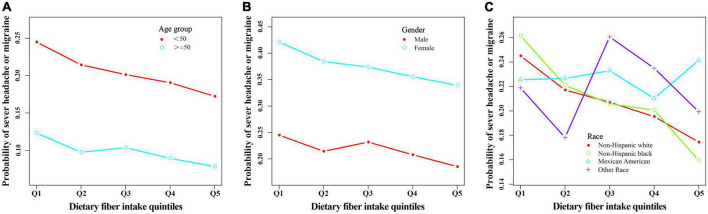
The relationship between headache or migraine with dietary fiber quintiles stratified by age **(A)**, gender **(B)**, and race **(C)** after adjusting related covariates in this study.

All statistical analyses were performed using EmpowerStats 2.2 (X&Y solutions Inc., Boston, MA, United States)^2^ and R (version 3.4.3).^3^ We considered *P*-values < 0.05 to be statistically significant. All reported *p*-values were two-sided. We calculated trend *P* by using the quintile of dietary fiber intake as an ordinal variable. Multivariable logistic regression models were constructed to evaluate odd ratios (ORs) with 95% confidence intervals (CIs) of severe headache or migraine at different levels of dietary fibers. Additionally, non-linear relationships were checked by fitting a generalized additive model (GAM) and smooth curve fitting between severe headaches or migraines and fiber intake quintiles (Figures 2, 3). In the main analysis and sensitivity analysis, since the status (“0” = no; “1” = yes) of severe headache or migraine were used as binary dependent variables and several covariates were adjusted, we used multivariable logistic regression. The lowest quintile of dietary fibers was considered as the reference group. No latent covariates were adjusted for the first model (univariable logistic regression); The second model was adjusted for age (smooth), gender, and race; The third model was further adjusted for age (smooth), BMI (smooth), C reactive protein (smooth), alcohol intake (smooth), fat intake (smooth), protein intake (smooth), total energy intake (smooth), family poverty income ratio (smooth), stroke, gender, hypertension history, physical activity, race, and smoked at least 100 cigarettes in life (Table 2). In addition, the GAM was used to adjust for several continuous variables marked as smooth in the equation to investigate the potential non-linearity relationship between these variables and migraine (12, 13). Furthermore, we performed subgroup analyses after stratifying for age, gender, and race.

We performed weighted and unweighted analyses using EmpowerStats. We chose to present only the unweighted results, because our weighted results were consistent with the unweighted estimates. Second, our purpose was not to produce national estimates, but to explore the association between severe headache or migraine and fiber intake. Third, there are different opinions about the proper use of the sample weights with the NHANES (14–16).

To reduce the effects of selection bias, propensity score matching (PSM) was performed using the statistical program EmpowerStats based on the variables in Supplementary Table 7. PSM method was used to run sensitivity analysis using a 0.05 caliper (Supplementary Table 7). For the matching, a 1:1 matching technique with replacement was utilized. In addition, we explored the potential for unmeasured confounding dietary between dietary fiber and severe headache or migraine by calculating *E*-values. The *e*-value quantifies the required magnitude of an unmeasured confounder that would negate the observed association between dietary fiber and severe headaches or migraine (17).

## Results

A total of 31,126 participants interviewed by NHANES between 1999 and 2004. We excluded subjects who were pregnant (*n* = 889) because pregnancy can alter dietary intake and body weight. We also excluded participants lack of data on migraines and headaches (*n* = 15,676), lack of data on dietary fiber intake (*n* = 1,847), and dietary fiber intake of more than 100 g per day (*n* = 4), leaving 12,710 subjects for the following analyses (Figure 1). Of these subjects, 19.9% (*n* = 2,527) experienced severe headaches or migraines. The main demographic and clinical characteristics of the subjects are shown in Table 1. Because of some baseline feature imbalance (7/14 baseline variables as covariates, *P* < 0.05) between included and excluded participants (Supplementary Table 8), PSM analysis was performed to assess potential selection bias. The findings after matching were consistent with the main results (Tables 2, 3 and Supplementary Tables 9, 10). According to dietary fiber intake, subjects were divided into quintiles: Q1 (0.0–7.79 g/day), Q2 (7.80–11.59 g/day), Q3 (11.60–15.70 g/day), Q4 (15.71–22.08 g/day), and Q5 (22.10–95.50 g/day). Overall, the incidence of severe headache or migraine decreased with increased intake of dietary fiber (24.0% for Q1, 21.0% for Q2, 19.5% for Q3, 18.1% for Q4, and 16.9% for Q5, respectively, Table 1).

**TABLE 1:** Demographics for the combined National Health and Nutrition Examination Survey (NHANES) 1999–2004 (raw data).

Fiber intake (g/day) quintiles	Q1 (0.00–7.79), *n* = 2,536	Q2 (7.80–11.59), *n* = 2,534	Q3 (11.60–15.70), *n* = 2,556	Q4 (15.71–22.08), *n* = 2,537	Q5 (22.10–95.50), *n* = 2,547	*P*-value
Age (years)	50.2 ± 18.7	51.6 ± 19.2	51.5 ± 19.2	51.7 ± 18.9	50.2 ± 18.1	0.001
Family poverty income ratio	2.3 ± 1.5	2.5 ± 1.6	2.7 ± 1.6	2.8 ± 1.6	2.8 ± 1.6	<0.001
C reactive protein, mg/dL	0.6 ± 1.1	0.5 ± 0.8	0.5 ± 1.1	0.4 ± 1.0	0.4 ± 0.8	<0.001
BMI (Kg/m^2^)	28.6 ± 6.6	28.6 ± 6.5	28.4 ± 6.1	28.4 ± 6.2	27.7 ± 5.8	<0.001
Total energy intake (Kcal/day)	1,399.4 ± 676.0	1,768.0 ± 702.6	2,070.3 ± 827.4	2,315.4 ± 908.8	2,873.8 ± 1,209.0	<0.001
Fat intake (g/day)	52.3 ± 31.3	66.9 ± 33.6	78.1 ± 40.0	85.9 ± 44.1	103.2 ± 57.6	<0.001
Protein intake (g/day)	54.7 ± 33.1	67.7 ± 31.9	77.3 ± 35.7	86.1 ± 38.3	107.4 ± 49.7	<0.001
Fiber intake (g/day)	5.1 ± 1.8	9.7 ± 1.1	13.6 ± 1.2	18.6 ± 1.8	31.3 ± 9.6	<0.001
Alcohol intake (g/day)	10.6 ± 33.3	8.1 ± 24.2	9.5 ± 26.1	10.8 ± 35.8	11.4 ± 36.3	0.002
Gender						<0.001
Male	1,076 (42.4%)	1,038 (41.0%)	1,217 (47.6%)	1,370 (54.0%)	1,664 (65.3%)	
Female	1,460 (57.6%)	1,496 (59.0%)	1,339 (52.4%)	1,167 (46.0%)	883 (34.7%)	
Race						<0.001
Non-Hispanic white	1,113 (43.9%)	1,272 (50.2%)	1,394 (54.5%)	1,437 (56.6%)	1,262 (49.5%)	
Non-Hispanic black	805 (31.7%)	577 (22.8%)	433 (16.9%)	364 (14.3%)	312 (12.2%)	
Mexican American	438 (17.3%)	484 (19.1%)	530 (20.7%)	571 (22.5%)	809 (31.8%)	
Other race	180 (7.1%)	201 (7.9%)	199 (7.8%)	165 (6.5%)	164 (6.4%)	
Stroke						<0.001
Yes	123 (4.9%)	103 (4.1%)	92 (3.6%)	87 (3.4%)	65 (2.6%)	
No	2,409 (95.1%)	2,430 (95.9%)	2,462 (96.4%)	2,446 (96.6%)	2,479 (97.4%)	
Smoke						<0.001
Yes	1,415 (55.9%)	1,240 (49.0%)	1,230 (48.2%)	1,198 (47.3%)	1,200 (47.2%)	
No	1,116 (44.1%)	1,291 (51.0%)	1,324 (51.8%)	1,336 (52.7%)	1,344 (52.8%)	
Hypertension history						<0.001
Yes	883 (35.2%)	886 (35.2%)	844 (33.3%)	821 (32.7%)	743 (29.6%)	
No	1,626 (64.8%)	1,628 (64.8%)	1,688 (66.7%)	1,686 (67.3%)	1,768 (70.4%)	
Physical activity						<0.001
Sits	736 (29.1%)	681 (26.9%)	690 (27.1%)	624 (24.6%)	539 (21.2%)	
Stands or walks	1,322 (52.2%)	1,406 (55.5%)	1,289 (50.5%)	1,307 (51.6%)	1,352 (53.1%)	
Light load	332 (13.1%)	328 (12.9%)	395 (15.5%)	421 (16.6%)	425 (16.7%)	
Heavy work	143 (5.6%)	118 (4.7%)	176 (6.9%)	181 (7.1%)	229 (9.0%)	
Headaches or migraines						<0.001
Yes	608 (24.0%)	532 (21.0%)	499 (19.5%)	458 (18.1%)	430 (16.9%)	
No	1,928 (76.0%)	2,002 (79.0%)	2,057 (80.5%)	2,079 (81.9%)	2,117 (83.1%)	

**TABLE 2:** Association between severe headache or migraine with fiber intake (raw data).

Exposure (OR (95% CI) *P*-value)	Model 1, *N* = 12,710	Model 2, *N* = 12,710	Model 3, *N* = 10,666
Fiber intake (10 g/day)	**0.86 (0.82, 0.90) <0.0001**	**0.91 (0.87, 0.96) 0.0002**	**0.89 (0.83, 0.95) 0.0005**
Fiber intake quintiles			
Q1	**1.0**	**1.0**	**1.0**
Q2	**0.84 (0.74, 0.96) 0.0111**	**0.86 (0.75, 0.98) 0.0294**	0.90 (0.77, 1.06) 0.2094
Q3	**0.77 (0.67, 0.88) 0.0001**	**0.82 (0.71, 0.94) 0.0053**	0.92 (0.78, 1.09) 0.3538
Q4	**0.70 (0.61, 0.80) <0.0001**	**0.78 (0.68, 0.90) 0.0007**	0.84 (0.71, 1.01) 0.0619
Q5	**0.64 (0.56, 0.74) <0.0001**	**0.74 (0.64, 0.86) <0.0001**	**0.74 (0.61, 0.90) 0.0029**
*P* for trend	**<0.0001**	**<0.0001**	**0.0044**

Model 1: No covariates were adjusted.

Model 2: Adjust for: Gender; age (Smooth), and race.

Model 3: Adjust for: age (Smooth); body mass index (BMI) (Smooth); C reactive protein (Smooth); alcohol intake (Smooth); fat intake (Smooth); protein intake (Smooth); total energy intake (Smooth); family poverty income ratio (Smooth); stroke; gender; hypertension history; physical activity; race; smoked at least 100 cigarettes in life. The bold values mean statistically different (*P* < 0.05) data.

**TABLE 3:** Association between severe headache or migraine with fiber intake quintiles stratified by gender, age, and race (raw data).

Stratification analysis	*N*	Fiber intake quintiles (OR (95% CI) *P*-value)					
		**Q1**	**Q2**	**Q3**	**Q4**	**Q5**	***P* for trend**
Gender							
Male	6,365	**1.0**	0.93 (0.70, 1.23) 0.5918	1.07 (0.81, 1.41) 0.6349	0.95 (0.71, 1.27) 0.7290	0.84 (0.61, 1.14) 0.2616	0.3087
Female	6,345	**1.0**	0.88 (0.72, 1.06) 0.1721	0.83 (0.67, 1.02) 0.0737	**0.77 (0.61, 0.96) 0.0217**	**0.66 (0.51, 0.86) 0.0021**	**0.0035**
Age (years)							
<50	6,275	**1.0**	0.90 (0.74, 1.10) 0.3148	0.87 (0.70, 1.07) 0.1926	**0.82 (0.65, 1.02) 0.0800**	**0.73 (0.57, 0.94) 0.0142**	**0.0137**
>=50	6,435	**1.0**	0.81 (0.63, 1.05) 0.1082	0.86 (0.66, 1.12) 0.2591	**0.72 (0.54, 0.95) 0.0223**	**0.59 (0.43, 0.82) 0.0016**	**0.0025**
Race							
Non-Hispanic white	6,478	**1.0**	0.93 (0.74, 1.18) 0.5470	0.90 (0.70, 1.14) 0.3780	0.83 (0.64, 1.07) 0.1422	**0.69 (0.52, 0.92) 0.0128**	**0.0082**
Non-Hispanic black	2,491	**1.0**	0.83 (0.61, 1.12) 0.2281	0.75 (0.53, 1.07) 0.1086	0.73 (0.49, 1.10) 0.1360	**0.54 (0.34, 0.87) 0.0110**	**0.0126**
Mexican American	2,832	**1.0**	1.00 (0.70, 1.43) 0.9998	1.01 (0.70, 1.45) 0.9564	0.91 (0.62, 1.33) 0.6303	1.07 (0.72, 1.61) 0.7367	0.9126
Other race	909	**1.0**	0.74 (0.41, 1.35) 0.3318	1.38 (0.76, 2.53) 0.2921	1.10 (0.56, 2.17) 0.7862	0.88 (0.41, 1.87) 0.7349	0.7603

Adjust for: age (Smooth); body mass index (BMI) (Smooth); C reactive protein (Smooth); alcohol intake (Smooth); fat intake (Smooth); protein intake (Smooth); total energy intake (Smooth); family poverty income ratio (Smooth); stroke; gender; hypertension history; physical activity; race; smoked at least 100 cigarettes in life. In the subgroup analysis stratified by gender, age, and race, the model is not adjusted for the stratification variable. The bold values mean statistically different (P < 0.05) data.

There was an inverse association between dietary fiber and the prevalence of severe headache or migraine. After adjusting for all covariates, we found that for every 10 g/day increase in dietary fiber intake, the prevalence of severe headache or migraine decreased by 11% (Table 2). In addition, in model 3, the risk of severe headache or migraine was reduced by 26% (95% CI, 0.61–0.90) in the Q5 group (the fifth quintile, highest dietary fiber intake) compared with Q1 group (the first quintile, lowest dietary fiber intake) (Table 2). The above results in the main analysis can be seen visually with smoothing curves (Figure 2). Sensitivity analyses (including a MI sensitivity analysis in Supplementary Table 2) were consistent with this result (Supplementary Tables 1, 2, 11). In particular, we noted that several variables in the original data had missing values (Supplementary Table 4). Therefore, the sample size of Model 3 (all-adjusted model) is smaller than that of Model 1 (Table 2 and Supplementary Table 1). Since there were no missing values for the participants’ age, gender, and race variables (Supplementary Table 4), Model 2 (partially-adjusted model) and model 1 have the same sample size (Table 2 and Supplementary Table 1).

When we conducted a stratified logistic regression analysis, we found the inverse association disappeared in Mexican Americans and other races (Table 3, Figure 3). Sensitivity analysis results support the credibility of this stratified result (Supplementary Tables 12, 13).

Moreover, although we also found the inverse association disappeared in male in stratified analyses (Table 3 and Supplementary Table 14), sensitivity analyses suggested that these results were unstable (Supplementary Table 12). Among males non-Hispanic blacks, Q5 (the fifth quintile, highest dietary fiber intake) had a 55% lower risk of severe headache or migraine (95% confidence interval 0.22–0.92) compared with Q1 (the first quintile, lowest dietary fiber intake) (Supplementary Table 12). Interestingly, we found a positive correlation between dietary fiber intake and the prevalence of severe headaches or migraines in female subjects of other races, which may be caused by accidental factors (Supplementary Table 12).

In addition, we performed a subgroup analysis of female age (reproductive age versus menopause). The results remained stable, showing an inverse association between fiber intake and migraine, independent of age in women (Supplementary Table 15). However, when we did stratified analyses of BMI, the results were not stable. Only individuals with a BMI of <25 or ≥ 30 had an inverse link between dietary fiber consumption and severe headache or migraine (Supplementary Table 16). This means that BMI may have had an impact on the results.

We also showed the basic information of participants with and without severe headache migraine (Supplementary Table 17). The association between severe headaches or migraine with related variables by univariate analysis is depicted in Supplementary Table 18.

Finally, we generated an *E*-value to assess the sensitivity to unmeasured confounding. The negative association between fiber and migraine was influenced only when the unmeasured covariates, including untested other nutrients, had hazard ratios greater than >1.6 for both *x* (dietary fiber) and *y* (migraine).

## Discussion

The purpose of this study was to explore the association between severe headaches or migraines and dietary fiber intake based on large-sample data. Our results show that the dietary fiber intake (10 g/day) and its quintiles were positively associated with the prevalence of severe headache or migraine in 12,710 US men and women after adjustment for potential confounders. To the best of our knowledge, no previous study has examined the relationship between dietary fiber and severe headache or migraine. In addition, our study has some advantages. First, we collected data from a large, representative national cohort, including 12,710 samples over a long time span from 1999 to 2004, which would yield more comprehensive and reliable results. Second, stratified analysis further found that race played a significant role in this negative correlation. Finally, various sensitivity analyses were performed to verify the stability of the results.

Migraine is a multidimensional disease that seriously affects people’s daily life. The prevention and treatment of migraine headaches have been challenging due to the limited effectiveness of existing drug and non-drug therapies (18). Scientists have been searching for a safe and effective control strategy. Migraine is influenced by genetic and environmental factors, and more and more studies show that the daily diet is closely related to the occurrence and relief of migraine (3). Dietary interventions also cost less and have fewer side effects than drug interventions. The results of dietary interventions for migraine are still controversial. But in general, most of the literature suggests that avoiding foods/ingredients/nutrients that cause migraines and consuming beneficial foods/ingredients/nutrients can help improve migraines. Several types of diets are thought to improve migraine through a range of mechanisms, including improvements in serotonergic dysfunction, neuronal excitability, mitochondrial function in the brain, neuroinflammation, hypothalamic function, and platelet aggregation (19). A review of the literature suggests that a ketogenic diet, a high folic acid diet, a low-fat diet, a modified Atkins diet, and a high omega-3/low omega-6 diet are significantly effective in improving migraine (19). On the other hand, there are many potential dietary triggers, such as chocolate, citrus fruits, nuts, ice cream, tomatoes, onions, dairy products, alcoholic beverages, caffeine, aspartame, and gluten (20, 21). In addition, certain foods trigger migraines depending on the amount and timing of consumption (20, 21).

The mechanisms by which dietary fiber acts on severe headaches or migraines are largely unknown, and there has been little research on their relationship. In recent years, the concept of gut-brain axis has attracted attention in several medical fields (10). Many studies have linked migraines to different gastrointestinal disorders, such as inflammatory bowel disease and celiac disease (22). Some researchers have speculated that the interaction between the gut and the brain may lead to neuro-related problems (10). Therefore, modulation of gut microbiota has been proposed to prevent and treat these problems. Although the mechanism of the influence of the gut-brain axis on migraine has not been explored, it has been suggested that increased intestinal permeability may lead to the arrival of pro-inflammatory substances in the trigeminal vascular system, leading to the occurrence of migraine (23). This theory is consistent with previous studies that have linked migraines to a variety of inflammatory diseases, such as allergies, with potential relationships involving inflammatory factors, gut microbiota, and nutritional elements (24). There is evidence that moderate increases in dietary fiber intake may be beneficial in regulating gut microbiota and the gut-brain axis, thereby improving migraines (25). Many studies have shown that gut bacteria can metabolize dietary fiber that cannot be digested by human enzymes, and thus produce microbial metabolites. Short chain fatty acids (SCFAs) are the main components of these metabolites, which are produced by microbial fermentation of specific dietary fiber. SCFAs provide the main energy source for colon cells, are essential for maintaining the integrity of the intestinal barrier, and have broad implications for immune and inflammatory regulation (26). Studies have shown that dietary fiber can affect the intestinal SCFAs level by affecting intestinal flora activity (27). Studies have shown that the natural short-chain fatty acids sodium butyrate and sodium propionate inhibit Histone Deacetylases (HDACs), which leads to the hyperacetylation of core histone proteins (H3 and H4) expressed by some genes associated with inflammation and the translocation of the well-known inflammatory mediator Nuclear Factor kappa-light-chain-enhancer of activated B cells (NF-B), which in turn decreases oxidative stress and the activation of the inflammatory cascade (28–30). In conclusion, dietary fiber may reduce inflammation and relieve migraine by regulating the gut microbiota and its metabolites, SCFAs.

The glycemic index of food decreases with the increase in dietary fiber content (31). There is countless evidence that a low glycemic diet (LGD) has a significant effect in improving conditions such as diabetes, obesity, hyperlipidemia, epilepsy, and so on. A LGD is generally considered to include carbohydrates with a glycemic index of less than 50 and is limited to 40–60 grams per day (32). Low glycemic index foods include vegetables, fruits, legumes, and whole grains, all of which are high in dietary fiber. A randomized controlled trial assigned 350 migraine patients 1:1 to either a LGD (considered as a high-fiber diet) group or prophylactic medication group. One month after the intervention, the frequency of migraine attacks was significantly reduced in both groups. The intensity of headache episodes was also significantly reduced in the LGD group 3 months after the intervention (31). The underlying mechanism by which a LGD improves headaches works, at least in part, by altering the inflammatory response. Studies have shown that a LGD can reduce soluble TNF-A receptor II and C-reactive protein (CRP) levels (33). In summary, the underlying mechanism by which dietary fiber can improve migraine may involve regulating intestinal flora and improving inflammatory response. What is more, vegetables, fruits, and legumes are rich in dietary fiber, and low intake of these foods in the first group (Q1 and Q2) may result in insufficient intake of certain vitamins, minerals, and antioxidants. It has been reported in the literature that appropriate intake of vitamins and micronutrients may play a key role in migraine prevention (34, 35). More studies, especially randomized controlled clinical studies, are needed in the future to confirm causality.

Worldwide, migraine is three times more common in women than in men. Estrogen plays a significant role in migraine, and it fluctuates with several life stages, including puberty, menstruation, pregnancy, and the peri- and post-menopausal years (36). So we did subgroup analyses based on women’s age and stage of childbearing (fertile age vs. menopause), and the results remained stable. The inverse association between dietary fiber intake and migraine was independent of women’s age. In addition, studies have shown that BMI is associated with migraine, with both underweight and obesity increasing the risk of migraine (37, 38). Further subgroup analysis of BMI showed stable results for participants with BMI <25 and >30, but unstable results for those with BMI between 25 and 30. This means that BMI may influence the negative association between dietary fiber and migraine. More studies are needed to confirm these results and explore the underlying mechanisms.

Nevertheless, this study has several limitations. First, due to the nature of cross-sectional observational studies, directional causality cannot be determined in this study. Second, although this study used a reliable 24-h dietary recall method to assess total food intake, recall bias or reporting bias was unavoidable. Third, as exposure and outcome data are self-reported, there is a misclassification bias. Fourth, although we used PSM to minimize the selection bias in sensitivity analysis, a significant difference was observed in some variables (Supplementary Tables 7, 8), especially age and sex. Therefore, participant selection bias was inevitable in this study. Fifth, the potential effects of all related chronic diseases or other unmeasured confounders could not be ruled out. Therefore, a longitudinal study is necessary. Finally, not all dietary fibers can be classified as prebiotics, and dietary fibers were not classified in this study.

## Conclusion

In conclusion, we found that increased dietary fiber intake was associated with a decreased incidence of migraine. However, more prospective studies should be conducted to confirm their association before dietary recommendations.

## Data availability statement

The original contributions presented in this study are included in the article/Supplementary material, further inquiries can be directed to the corresponding author.

## Author contributions

KH and HH designed and conducted the research, performed the computational analysis, and contributed to the preparation of the manuscript. HH analyzed and interpreted the data and assisted with processing images. KH wrote the manuscript. Both authors contributed to editing and reviewing, read and approved the manuscript.

## Footnotes

1
http://www.cdc.gov/nchs/nhanes.htm

2
http://www.empowerstats.com

3
https://www.R-project.org
