# Supplementary material for: The association between dietary fiber intake and severe headaches or migraine in US adults
Source: Front Nutr. 2023 Jan 4;9:1044066. doi: 10.3389/fnut.2022.1044066 (PMC9846638; doi:10.3389/fnut.2022.1044066)
Supplement: Supplementary file 1 [file Data_Sheet_1.docx]

***Supplementary Material***

**Supplementary Table 1.** Association between severe headache or migraine with fiber intake (two-day average) from NHANES 2003-2004 (raw data)

| **Exposure (OR (95%CI) P-value)** | **Model 1,**  **N=3,927** | **Model 2,**  **N=3,927** | **Model 3,**  **N=3,479** |
| --- | --- | --- | --- |
| **Fiber intake, 10 g/d** | **0.75 (0.68, 0.84) <0.0001** | **0.79 (0.71, 0.89) <0.0001** | **0.72 (0.61, 0.84) <0.0001** |
| **Fiber intake** **quintiles** |  |  |  |
| Q1 | **1.0** | **1.0** | **1.0** |
| Q2 | 0.80 (0.63, 1.01) 0.0552 | 0.81 (0.64, 1.04) 0.0982 | 0.83 (0.63, 1.09) 0.1815 |
| Q3 | **0.71 (0.56, 0.90) 0.0049** | **0.76 (0.60, 0.98) 0.0314** | 0.79 (0.59, 1.06) 0.1138 |
| Q4 | **0.57 (0.44, 0.72) <0.0001** | **0.63 (0.49, 0.82) 0.0005** | **0.59 (0.43, 0.82) 0.0015** |
| Q5 | **0.51 (0.40, 0.66) <0.0001** | **0.57 (0.44, 0.75) <0.0001** | **0.48 (0.33, 0.68) <0.0001** |
| P for trend | **<0.0001** | **<0.0001** | **<0.0001** |

Model 1: No covariates were adjusted.

Model 2: Adjust for: Gender; Age (Smooth), Race.

Model 3: Adjust for: Age (Smooth); BMI (Smooth); C reactive protein (Smooth); Alcohol intake (two-day average, Smooth); Fat intake (two-day average, Smooth); Protein intake (two-day average, Smooth); Total energy intake (two-day average, Smooth); Family poverty income ratio (Smooth); Stroke; Gender; Hypertension history; Physical activity; Race; Smoked at least 100 cigarettes in life.

**Supplementary Table 2.** Association between severe headache or migraine with Fiber intake (5 imputed datasets, and their pooled).

| **Datasets** | **OR** | **95%CI** | **P-value** | **P for trend** |
| --- | --- | --- | --- | --- |
| **Dataset 1, n= 12,710** |  |  |  |  |
| **Fiber intake, 10g/d** | **0.89** | **0.83, 0.94** | **0.0001** |  |
| **Fiber intake** **quintiles** |  |  |  |  |
| Q1 | **1.0** |  |  | **0.0007** |
| Q2 | 0.90 | 0.78, 1.04 | 0.1494 |  |
| Q3 | 0.86 | 0.74, 1.01 | 0.0651 |  |
| Q4 | **0.82** | **0.70, 0.97** | **0.0176** |  |
| Q5 | **0.73** | **0.60, 0.87** | **0.0006** |  |
| **Dataset 2, n= 12,710** |  |  |  |  |
| **Fiber intake, 10g/d** | **0.88** | **0.83, 0.94** | **0.0001** |  |
| **Fiber intake** **quintiles** |  |  |  | **0.0006** |
| Q1 | **1.0** |  |  |  |
| Q2 | 0.90 | 0.78, 1.04 | 0.1545 |  |
| Q3 | 0.86 | 0.74, 1.00 | 0.0564 |  |
| Q4 | **0.82** | **0.69, 0.97** | **0.0171** |  |
| Q5 | **0.72** | **0.60, 0.87** | **0.0005** |  |
| **Dataset 3, n= 12,710** |  |  |  |  |
| **Fiber intake, 10g/d** | **0.89** | **0.83, 0.94** | **0.0001** |  |
| **Fiber intake** **quintiles** |  |  |  | **0.0007** |
| Q1 | **1.0** |  |  |  |
| Q2 | 0.90 | 0.77, 1.04 | 0.1391 |  |
| Q3 | 0.86 | 0.74, 1.01 | 0.0586 |  |
| Q4 | **0.82** | **0.70, 0.97** | **0.0183** |  |
| Q5 | **0.72** | **0.60, 0.87** | **0.0006** |  |
| **Dataset 4, n= 12,710** |  |  |  |  |
| **Fiber intake, 10g/d** | **0.88** | **0.83, 0.94** | **<0.0001** |  |
| **Fiber intake** **quintiles** |  |  |  |  |
| Q1 | **1.0** |  |  | **0.0006** |
| Q2 | 0.90 | 0.78, 1.04 | 0.1438 |  |
| Q3 | 0.86 | 0.74, 1.00 | 0.0527 |  |
| Q4 | **0.82** | **0.70, 0.97** | **0.0179** |  |
| Q5 | **0.72** | **(0.60, 0.87** | **0.0005** |  |
| **Dataset 5, n= 12,710** |  |  |  |  |
| **Fiber intake, 10g/d** | **0.88** | **0.83, 0.94** | **<0.0001** |  |
| **Fiber intake** **quintiles** |  |  |  | **0.0006** |
| Q1 | **1.0** |  |  |  |
| Q2 | 0.90 | 0.77, 1.04 | 0.1406 |  |
| Q3 | 0.86 | 0.74, 1.00 | 0.0554 |  |
| Q4 | **0.82** | **0.69, 0.96** | **0.0165** |  |
| Q5 | **0.72** | **0.60, 0.87** | **0.0005** |  |
| **Pooled Dataset 1-5, n= 12,710** |  |  |  |  |
| **Fiber intake, 10g/d** | **0.88** | **0.83, 0.94** | **0.0001** |  |
| **Fiber intake** **quintiles** |  |  |  | **0.0010** |
| Q1 | **1.0** |  |  |  |
| Q2 | 0.90 | 0.78, 1.04 | 0.1585 |  |
| Q3 | 0.86 | 0.74, 1.00 | 0.0527 |  |
| Q4 | **0.82** | **0.70, 0.97** | **0.0184** |  |
| Q5 | **0.72** | **0.60, 0.87** | **0.0006** |  |

Adjust for: Age (Smooth); BMI (Smooth); C reactive protein (Smooth); Alcohol intake (Smooth); Fat intake (Smooth); Protein intake (Smooth); Total energy intake (Smooth); Family poverty income ratio (Smooth); Stroke; Gender; Hypertension history; Physical activity; Race; Smoked at least 100 cigarettes in life.

**Supplementary Table 3.** Imputed data summary (pooled 1-5 imputed datasets)

|  | **Min.** | **1st Qu.** | **Median** | **Mean** | **3rd Qu.** | **Max.** |
| --- | --- | --- | --- | --- | --- | --- |
| **Race** | 1 | 1 | 1 | 1.86 | 3 | 4 |
| **Gender** | 1 | 1 | 1 | 1.5 | 2 | 2 |
| **Stroke** | 1 | 2 | 2 | 1.96 | 2 | 2 |
| **Smoked at least 100 cigarettes in life** | 1 | 1 | 2 | 1.51 | 2 | 2 |
| **Physical activity** | 1 | 1 | 2 | 2.03 | 2 | 4 |
| **Hypertension history** | 1 | 1 | 2 | 1.67 | 2 | 2 |
| **Protein intake** | 0 | 50.48 | 71.41 | 78.68 | 97.59 | 718.42 |
| **Fat intake** | 0 | 45.62 | 68.25 | 77.31 | 98.88 | 839.65 |
| **Family poverty income ratio** | 0 | 1.19 | 2.23 | 2.62 | 4.11 | 48.06 |
| **Energy intake** | 0 | 1389.95 | 1901 | 2086.12 | 2583.24 | 15594 |
| **C reactive protein** | 0 | 0.09 | 0.23 | 0.48 | 0.51 | 29.6 |
| **BMI** | 9.84 | 24.07 | 27.42 | 28.36 | 31.49 | 66.44 |
| **Age** | 20 | 35 | 50 | 51.06 | 66 | 85 |
| **Alcohol intake** | 0 | 0 | 0 | 10.09 | 0.05 | 939 |

**Supplementary Table 4.** Missing data patterns (raw data).

| **Pattern** | **N** |
| --- | --- |
| **Nothing** | 10,666 |
| **Family poverty income ratio** | 964 |
| **BMI** | 264 |
| **C reactive protein** | 473 |
| **Hypertension history** | 107 |
| **Stroke** | 12 |
| **Physical activity** | 8 |
| **Smoked at least 100 cigarettes in life** | 9 |
| **Hypertension history, Family poverty income ratio** | 11 |
| **Family poverty income ratio, C reactive protein** | 84 |
| **Family poverty income ratio, BMI** | 17 |
| **Hypertension history, BMI** | 4 |
| **C reactive protein, BMI** | 52 |
| **Smoked at least 100 cigarettes in life, Family poverty income ratio** | 3 |
| **Smoked at least 100 cigarettes in life, Physical activity** | 1 |
| **Physical activity, family poverty income ratio** | 3 |
| **Hypertension history, C reactive protein** | 8 |
| **Physical activity, BMI** | 1 |
| **Stroke, Family poverty income ratio** | 1 |
| **Physical activity, C reactive protein** | 1 |
| **Stroke, C reactive protein** | 1 |
| **Smoked at least 100 cigarettes in life, BMI** | 1 |
| **Hypertension history, c reactive protein, BMI** | 2 |
| **Hypertension history, Family poverty income ratio, C reactive protein** | 4 |
| **Family poverty income ratio, C reactive protein, BMI** | 9 |
| **Physical activity, Family poverty income ratio, C reactive protein** | 1 |
| **Smoked at least 100 cigarettes in life, Family poverty income ratio, BMI** | 1 |
| **Smoked at least 100 cigarettes in life, Physical activity, Family poverty income ratio** | 1 |
| **Hypertension history, Family poverty income ratio, BMI** | 1 |

**Supplementary Table 5.** Raw data summary (raw data)

|  | **Min.** | **1st Qu.** | **Median** | **Mean** | **3rd Qu.** | **Max.** |
| --- | --- | --- | --- | --- | --- | --- |
| **Race** | 1 | 1 | 1 | 1.86 | 3 | 4 |
| **Gender** | 1 | 1 | 1 | 1.5 | 2 | 2 |
| **Stroke** | 1 | 2 | 2 | 1.96 | 2 | 2 |
| **Smoked at least 100 cigarettes in life** | 1 | 1 | 2 | 1.51 | 2 | 2 |
| **Physical activity** | 1 | 1 | 2 | 2.03 | 2 | 4 |
| **Hypertension history** | 1 | 1 | 2 | 1.67 | 2 | 2 |
| **Protein intake** | 0 | 50.48 | 71.41 | 78.68 | 97.59 | 718.42 |
| **Fat intake** | 0 | 45.62 | 68.25 | 77.31 | 98.88 | 839.65 |
| **Family poverty income ratio** | 0 | 1.2 | 2.26 | 2.61 | 4.16 | 5 |
| **Energy intake** | 0 | 1389.96 | 1901 | 2086.12 | 2583.11 | 15594 |
| **C reactive protein** | 0.01 | 0.09 | 0.23 | 0.48 | 0.51 | 29.6 |
| **BMI** | 12.04 | 24.06 | 27.39 | 28.33 | 31.44 | 66.44 |
| **Age** | 20 | 35 | 50 | 51.06 | 66 | 85 |
| **Alcohol intake** | 0 | 0 | 0 | 10.09 | 0.05 | 939 |

**Supplementary Table 6.** Variables description and coding.

| **Variable** | **Label in data** | **Description** | **Code or value** |
| --- | --- | --- | --- |
| Datasets | mi.iter | Categorical variables | 0 = Raw dataset (n=34102)  1-5 = Dataset 1-5 (using multiple imputation) |
| ID | SEQN_ID | Categorical variables |  |
| Gender | GENDR | Categorical variables | 1 = male  2 = female |
| Race | RACE | Categorical variables | 1 = Non-Hispanic White  2 = Non-Hispanic Black  3 = Mexican American  4 = Other Race |
| Stroke | STROKE | Categorical variables | 1 = Yes  0 = N0 |
| Hypertension history | HYPERTESION | Categorical variables | 1 = Yes  2 = N0 |
| Stroke |  | Categorical variables | 1 = Yes  2 = N0 |
| Physical activity | PHYSICAL | Categorical variables | 1 = sits  2= stands or walks  3 = light load  4 = heavy work |
| Smoked at least 100 cigarettes in life | SMOKED.A | Categorical variables | 1 = Yes  2 = N0 |
| Headache or migraine | HEADACHE | Categorical variables | 1 = Yes  0 = N0 |
| Age | AGE | Continuous variables |  |
| Age groups | AGE.RCD | Categorical variables | 1 = <50 years  2 = >=50 years |
| Family poverty income ratio | FAMILYPIR | Continuous variables |  |
| C reactive protein | C.REACTIVE.P | Continuous variables |  |
| BMI | BMI | Continuous variables |  |
| BMI groups | BMI.RCD | Categorical variables | 1= <25  2= >=25, <30  3= >=30 |
| BMI tertiles | BMI.RCD1 | Categorical variables | 1 = Low  2 = Middle  3 = High |
| Total energy intake | ENERGY | Continuous variables |  |
| Fiber intake | NEWFIBER | Continuous variables |  |
| Alcohol intake | NEWALCOHOL | Continuous variables |  |
| Fat intake | NEWFAT | Continuous variables |  |
| Protein intake | NEWPROTEIN | Continuous variables |  |
| Fiber intake | NEWFIBER | Continuous variables |  |
| Fiber intake dichotomous | FIBER dichotomous | Categorical variables | 1 = Low  2 = High |
| Fiber intake tertiles | FIBER tertiles | Categorical variables | 1 = Low  2 = Middle  3 = High |
| Fiber intake quartiles | FIBER quartiles | Categorical variables | 1 = Q1  2 = Q2  3 = Q3  4 = Q4 |
| Fiber intake quartiles | FIBER quartiles | Categorical variables | 1 = Q1  2 = Q2  3 = Q3  4 = Q4  5 = Q5 |

**Supplementary Table 7.** Characteristic of included participants’ baseline before and after propensity score matching (raw data).

| **Datasets** | **Before** **matching** |  |  | **After matching** |  |  |
| --- | --- | --- | --- | --- | --- | --- |
|  | **Headaches or migraines, No**  **n=10,183** | **Headaches or migraines, Yes**  **n=2,527** | **P-value*** | **Headaches or migraines, No**  **n=2,146** | **Headaches or migraines, Yes**  **n=2,146** | **P-value*** |
| **Age, years** | 52.6 ± 19.1 | 45.0 ± 16.2 | <0.001 | 48.9 ± 18.1 | 44.5 ± 15.8 | <0.001 |
| **Family poverty income ratio** | 2.7 ± 1.6 | 2.3 ± 1.5 | <0.001 | 2.6 ± 1.6 | 2.3 ± 1.5 | <0.001 |
| **C reactive protein, mg/dL** | 0.5 ± 1.0 | 0.5 ± 0.8 | <0.001 | 0.5 ± 1.18 | 0.5 ± 0.8 | 0.083 |
| **BMI, Kg/m^2^** | 28.2 ± 6.0 | 28.9 ± 6.9 | <0.001 | 28.7 ± 6.6 | 28.9 ± 6.9 | 0.227 |
| **Total energy intake, Kcal/d** | 2091.4 ± 1007.5 | 2064.9 ± 1053.8 | 0.049 | 2101.2 ± 1096.4 | 2094.6 ± 1062.0 | 0.795 |
| **Fat intake, g/d** | 77.6 ± 45.2 | 76.1 ± 47.6 | 0.014 | 77.4 ± 47.0 | 77.9 ± 48.5 | 0.752 |
| **Protein intake, g/d** | 79.3 ± 41.6 | 76.2 ± 44.4 | <0.001 | 78.0 ± 42.0 | 76.9 ± 44.3 | 0.176 |
| **Alcohol intake, g/d** | 10.8 ± 33.1 | 7.1 ± 23.8 | <0.001 | 10.5 ± 44.0 | 7. 2 ± 23.8 | <0.001 |
| **Gender** |  |  | <0.001 |  |  | <0.001 |
| Male | 5485 (53.9%) | 880 (34.8%) |  | 938 (43.7%) | 753 (35.1%) |  |
| Female | 4698 (46.1%) | 1647 (65.2%) |  | 1208 (56.3%) | 1393 (64.9%) |  |
| **Race** |  |  | <0.001 |  |  | 0.081 |
| Non-Hispanic white | 5320 (52.2%) | 1158 (45.8%) |  | 1098 (51.2%) | 1019 (47.5%) |  |
| Non-Hispanic black | 1950 (19.1%) | 541 (21.4%) |  | 424 (19.8%) | 447 (20.8%) |  |
| Mexican American | 2218 (21.8%) | 614 (24.3%) |  | 480 (22.4%) | 509 (23.7%) |  |
| Other Race | 695 (6.8%) | 214 (8.5%) |  | 144 (6.7%) | 171 (8.0%) |  |
| **Stroke** |  |  | 0.028 |  |  | 0.108 |
| Yes | 358 (3.5%) | 112 (4.4%) |  | 63 (2.9%) | 82 (3.8%) |  |
| No | 9816 (96.5%) | 2410 (95.6%) |  | 2083 (97.1%) | 2064 (96.2%) |  |
| **Smoke** |  |  | 0.511 |  |  | 0.313 |
| Yes | 5048 (49.6%) | 1235 (48.9%) |  | 1005 (46.8%) | 1038 (48.4%) |  |
| No | 5121 (50.4%) | 1290 (51.1%) |  | 1141 (53.2%) | 1108 (51.6%) |  |
| **Hypertension**  **history** |  |  | 0.617 |  |  | 0.922 |
| Yes | 3355 (33.3%) | 822 (32.8%) |  | 694 (32.3%) | 697 (32.5%) |  |
| No | 6712 (66.7%) | 1684 (67.2%) |  | 1452 (67.7%) | 1449 (67.5%) |  |
| **Physical activity** |  |  | 0.011 |  |  | 0.068 |
| sits | 2555 (25.1%) | 715 (28.3%) |  | 532 (24.8%) | 605 (28.2%) |  |
| stands or walks | 5396 (53.1%) | 1280 (50.7%) |  | 1164 (54.2%) | 1091 (50.8%) |  |
| light load | 1540 (15.1%) | 361 (14.3%) |  | 312 (14.5%) | 308 (14.4%) |  |
| Heavy work | 676 (6.6%) | 171 (6.8%) |  | 138 (6.4%) | 142 (6.6%) |  |

*Kruskal−Wallis test

**Supplementary Table 8.** Baseline characteristics of excluded (missing values of migraine and fiber intake) and included participants (raw data).

| **Variable** | **Included particapants n=1,2710** | **Excluded particapants n=1,858** | **P-value*** |
| --- | --- | --- | --- |
| **Age, years** | 51.1 ± 18.8 | 55.5 ± 21.6 | <0.001 |
| **Family poverty income ratio** | 2.6 ± 1.6 | 2.5 ± 1.6 | 0.045 |
| **C reactive protein, mg/dL** | 0.5 ± 1.0 | 0.6 ± 1.3 | 0.078 |
| **BMI, Kg/m^2^** | 28.3 ± 6.2 | 27.4 ± 5.6 | <0.001 |
| **Total energy intake, Kcal/d** | 2086.1 ± 1016.9 | 2119.8 ± 988.5 | 0.810 |
| **Fat intake, g/d** | 77.3 ± 45.7 | 76.8 ± 56.9 | 0.643 |
| **Protein intake, g/d** | 78.7 ± 42.2 | 71.0 ± 41.5 | 0.768 |
| **Alcohol intake, g/d** | 10.1 ± 31.5 | 0.0 ± 0.0 | 0.153 |
| **Gender** |  |  | 0.001 |
| Male | 6365 (50.1%) | 855 (46.0%) |  |
| Female | 6345 (49.9%) | 1003 (54.0%) |  |
| **Race** |  |  | <0.001 |
| Non-Hispanic white | 6478 (51.0%) | 975 (52.5%) |  |
| Non-Hispanic black | 2491 (19.6%) | 364 (19.6%) |  |
| Mexican American | 2832 (22.3%) | 341 (18.4%) |  |
| Other Race | 909 (7.2%) | 178 (9.6%) |  |
| **Stroke** |  |  | <0.001 |
| Yes | 470 (3.7%) | 136 (7.3%) |  |
| No | 12226 (96.3%) | 1715 (92.7%) |  |
| **Smoke** |  |  | 0.079 |
| Yes | 6283 (49.5%) | 870 (47.3%) |  |
| No | 6411 (50.5%) | 969 (52.7%) |  |
| **Hypertension history** |  |  | 0.465 |
| Yes | 4177 (33.2%) | 618 (34.1%) |  |
| No | 8396 (66.8%) | 1195 (65.9%) |  |
| **Physical activity** |  |  | <0.001 |
| sits | 3270 (25.8%) | 651 (35.3%) |  |
| stands or walks | 6676 (52.6%) | 845 (45.8%) |  |
| light load | 1901 (15.0%) | 217 (11.8%) |  |
| Heavy work | 847 (6.7%) | 130 (7.1%) |  |

*Kruskal−Wallis test

**Supplementary Table 9.** Association between severe headache or migraine with fiber intake among included participants before and after propensity score matching (raw data).

| **Fiber intake quintiles (OR (95%CI) P-value)** | **Before matching** |  |  | **After matching** |  |  |
| --- | --- | --- | --- | --- | --- | --- |
|  | **Model 1,**  **N=12,710** | **Model 2,**  **N=12,710** | **Model 3,**  **N=10,666** | **Model 1,**  **N=4,292** | **Model 2,**  **N=4,292** | **Model 3,**  **N=4,292** |
| Q1 | **1.0** | **1.0** | 1.0 | **1.0** | 1.0 | 1.0 |
| Q2 | **0.84 (0.74, 0.96) 0.0111** | **0.86 (0.75, 0.98) 0.0294** | 0.90 (0.77, 1.06) 0.2094 | **0.80 (0.67, 0.96) 0.0173** | **0.82 (0.68, 0.99) 0.0430** | **0.81 (0.67, 0.99) 0.0402** |
| Q3 | **0.77 (0.67, 0.88) 0.0001** | **0.82 (0.71, 0.94) 0.0053** | 0.92 (0.78, 1.09) 0.3538 | **0.89 (0.73, 1.07) 0.2062** | 0.93 (0.77, 1.12) 0.4376 | 0.89 (0.72, 1.10) 0.2944 |
| Q4 | **0.70 (0.61, 0.80) <0.0001** | **0.78 (0.68, 0.90) 0.0007** | 0.84 (0.71, 1.01) 0.0619 | **0.77 (0.64, 0.94) 0.0084** | 0.82 (0.68, 1.00) 0.0510 | **0.78 (0.62, 0.98) 0.0350** |
| Q5 | **0.64 (0.56, 0.74) <0.0001** | **0.74 (0.64, 0.86) <0.0001** | **0.74 (0.61, 0.90) 0.0029** | **0.65 (0.54, 0.79) <0.0001** | **0.71 (0.58, 0.86) 0.0006** | **0.65 (0.51, 0.83) 0.0005** |
| P for trend | **<0.0001** | **<0.0001** | **0.0044** | **<0.0001** | **0.0024** | **0.0019** |

Model 1: No covariates were adjusted.

Model 2: Adjust for: Gender; Age (Smooth), Race.

Model 3: Adjust for: Age (Smooth); BMI (Smooth); C reactive protein (Smooth); Alcohol intake (Smooth); Fat intake (Smooth); Protein intake (Smooth); Total energy intake (Smooth); Family poverty income ratio (Smooth); Stroke; Gender; Hypertension history; Physical activity; Race; Smoked at least 100 cigarettes in life.

**Supplementary Table 10.** Association between severe headache or migraine with fiber intake quintiles stratified by gender, age, and race after propensity score matching (raw data).

| **Stratification analysis** | **N** | **Fiber intake quintiles (OR (95%CI) P-value)** | | | | | |
| --- | --- | --- | --- | --- | --- | --- | --- |
|  |  | **Q1** | **Q2** | **Q3** | **Q4** | **Q5** | **P for trend** |
| **Gender** |  |  |  |  |  |  |  |
| Male | 1,691 | **1.0** | 0.95 (0.66, 1.35) 0.7562 | 1.23 (0.86, 1.75) 0.2546 | 1.09 (0.76, 1.57) 0.6330 | 0.84 (0.57, 1.22) 0.3575 | 0.4461 |
| Female | 2,601 | **1.0** | **0.73 (0.58, 0.93) 0.0111** | **0.74 (0.57, 0.96) 0.0219** | **0.66 (0.50, 0.88) 0.0051** | **0.57 (0.41, 0.80) 0.0010** | **0.0016** |
| **Age, years** |  |  |  |  |  |  |  |
| <50 | 2,582 | **1.0** | 0.85 (0.66, 1.10) 0.2208 | 0.89 (0.68, 1.16) 0.3944 | 0.81 (0.61, 1.08) 0.1580 | **0.66 (0.48, 0.90) 0.0082** | **0.0156** |
| >=50 | 1,710 | **1.0** | **0.71 (0.52, 0.97) 0.0327** | 0.87 (0.62, 1.22) 0.4151 | **0.69 (0.48, 0.99) 0.0415** | **0.53 (0.35, 0.79) 0.0019** | **0.0083** |
| **Race** |  |  |  |  |  |  |  |
| Non-Hispanic white | 2,117 | **1.0** | 0.77 (0.58, 1.04) 0.0865 | 0.84 (0.62, 1.14) 0.2669 | **0.75 (0.54, 1.04) 0.0838** | **0.61 (0.42, 0.87) 0.0060** | **0.0161** |
| Non-Hispanic black | 871 | **1.0** | 0.82 (0.56, 1.20) 0.2966 | 0.70 (0.44, 1.10) 0.1226 | 0.79 (0.46, 1.35) 0.3886 | **0.49 (0.28, 0.88) 0.0171** | **0.0317** |
| Mexican American | 989 | **1.0** | 1.03 (0.65, 1.63) 0.8920 | 1.13 (0.71, 1.78) 0.6115 | 0.90 (0.56, 1.46) 0.6762 | 0.97 (0.59, 1.61) 0.9152 | 0.7245 |
| Other Race | 315 | **1.0** | 0.67 (0.32, 1.43) 0.2996 | 1.71 (0.78, 3.78) 0.1809 | 1.21 (0.50, 2.90) 0.6757 | 0.96 (0.35, 2.61) 0.9376 | 0.4699 |

Adjust for: Age (Smooth); BMI (Smooth); C reactive protein (Smooth); Alcohol intake (Smooth); Fat intake (Smooth); Protein intake (Smooth); Total energy intake (Smooth); Family poverty income ratio (Smooth); Stroke; Gender; Hypertension history; Physical activity; Race; Smoked at least 100 cigarettes in life.

**Note:** In the subgroup analysis stratified by gender, age, and race, the model is not adjusted for the stratification variable

**Supplementary Table 11.** Association between severe headache or migraine with fiber intake dichotomous, tertiles and quartiles (raw data).

| **Exposure (OR (95%CI) P-value)** | **Model 1,**  **N=12,710** | **Model 2,**  **N=12,710** | **Model 3,**  **N=10,**6**66** |
| --- | --- | --- | --- |
| **Fiber intake dichotomous** |  |  |  |
| Low | **1.0** | **1.0** | **1.0** |
| High | **0.76 (0.70, 0.83) <0.0001** | **0.84 (0.77, 0.92) 0.0003** | **0.88 (0.78, 0.98) 0.0263** |
| P for trend | **<0.0001** | **0.0003** | **0.0263** |
| **Fiber intake tertiles** |  |  |  |
| Low | **1.0** | **1.0** | **1.0** |
| Middle | **0.78 (0.70, 0.86) <0.0001** | **0.83 (0.75, 0.93) 0.0009** | 0.91 (0.80, 1.03) 0.1439 |
| High | **0.70 (0.63, 0.78) <0.0001** | **0.79 (0.71, 0.89) <0.0001** | **0.82 (0.70, 0.95) 0.0080** |
| P for trend | **<0.0001** | **<0.0001** | **0.0080** |
| **Fiber intake quartiles** |  |  |  |
| Q1 | **1.0** | **1.0** | **1.0** |
| Q2 | **0.82 (0.73, 0.92) 0.0009** | **0.84 (0.74, 0.95) 0.0062** | 0.91 (0.79, 1.05) 0.2111 |
| Q3 | **0.69 (0.61, 0.78) <0.0001** | **0.76 (0.67, 0.86) <0.0001** | **0.84 (0.72, 0.98) 0.0266** |
| Q4 | **0.69 (0.61, 0.78) <0.0001** | **0.79 (0.70, 0.90) 0.0005** | **0.81 (0.68, 0.97) 0.0202** |
| P for trend | **<0.0001** | **<0.0001** | **0.0123** |

Model 1: No covariates were adjusted.

Model 2: Adjust for: Gender; Age (Smooth), Race.

Model 3: Adjust for: Age (Smooth); BMI (Smooth); C reactive protein (Smooth); Alcohol intake (Smooth); Fat intake (Smooth); Protein intake (Smooth); Total energy intake (Smooth); Family poverty income ratio (Smooth); Stroke; Gender; Hypertension history; Physical activity; Race; Smoked at least 100 cigarettes in life.

**Supplementary Table 12.** Association between severe headache or migraine with fiber intake stratified by gender among different races (raw data).

| **Stratified by gender** | **N** | **Race tertiles (OR (95%CI) P-value)** | | | |
| --- | --- | --- | --- | --- | --- |
|  |  | **Non-Hispanic white** | **Non-Hispanic black** | **Mexican American** | **Other race** |
| **Male** |  |  |  |  |  |
| Q1 | 1,076 | **1.0** | **1.0** | **1.0** | **1.0** |
| Q2 | 1,038 | 0.86 (0.57, 1.31) 0.4951 | 0.74 (0.43, 1.30) 0.3026 | 1.41 (0.71, 2.80) 0.3320 | 0.54 (0.16, 1.81) 0.3215 |
| Q3 | 1,217 | 1.02 (0.68, 1.53) 0.9228 | 0.89 (0.51, 1.57) 0.6898 | 1.28 (0.64, 2.56) 0.4834 | 1.00 (0.33, 3.03) 0.9944 |
| Q4 | 1,370 | 0.92 (0.60, 1.40) 0.6974 | 0.79 (0.43, 1.43) 0.4316 | 1.11 (0.55, 2.24) 0.7634 | 0.72 (0.21, 2.42) 0.5952 |
| Q5 | 1,664 | 0.80 (0.51, 1.26) 0.3373 | **0.45 (0.22, 0.92) 0.0277** | 1.44 (0.71, 2.94) 0.3167 | 0.85 (0.24, 3.02) 0.8007 |
| P for trend | 6,365 | 0.4348 | 0.0779 | 0.5832 | 0.9890 |
| **Female** |  |  |  |  |  |
| Q1 | 1,460 | **1.0** | **1.0** | **1.0** | **1.0** |
| Q2 | 1,496 | 0.89 (0.67, 1.19) 0.4330 | 0.85 (0.59, 1.23) 0.3869 | 0.88 (0.57, 1.36) 0.5720 | 1.06 (0.50, 2.26) 0.8695 |
| Q3 | 1,339 | 0.75 (0.55, 1.02) 0.0628 | 0.67 (0.42, 1.05) 0.0794 | 0.92 (0.59, 1.44) 0.7246 | **2.22 (1.02, 4.83) 0.0450** |
| Q4 | 1,167 | **0.72 (0.52, 1.00) 0.0474** | 0.68 (0.39, 1.20) 0.1845 | 0.88 (0.55, 1.40) 0.5800 | 1.92 (0.75, 4.90) 0.1732 |
| Q5 | 883 | **0.57 (0.38, 0.85) 0.0053** | 0.63 (0.33, 1.21) 0.1688 | 0.94 (0.56, 1.58) 0.8211 | 0.77 (0.26, 2.28) 0.6406 |
| P for trend | 6,345 | **0.0028** | 0.0664 | 0.8455 | 0.6026 |

Adjust for: Age (Smooth); BMI (Smooth); C reactive protein (Smooth); Alcohol intake (Smooth); Fat intake (Smooth); Protein intake (Smooth); Total energy intake (Smooth); Family poverty income ratio (Smooth); Stroke; Hypertension history; Physical activity; Smoked at least 100 cigarettes in life.

**Supplementary Table 13.** Association between severe headache or migraine with fiber intake stratified by age groups among different races (raw data).

| **Stratified by** Age group**s** | **N** | Race **(OR (95%CI) P-value)** | | | |
| --- | --- | --- | --- | --- | --- |
|  |  | **Non-Hispanic white** | **Non-Hispanic black** | **Mexican American** | **Other race** |
| **<50 years** |  |  |  |  |  |
| **Q1** | 1,287 | **1.0** | **1.0** | **1.0** | **1.0** |
| **Q2** | 1,211 | 0.96 (0.701, 1.304) 0.7766 | 0.85 (0.579, 1.260) 0.4270 | 1.06 (0.660, 1.695) 0.8141 | 0.49 (0.223, 1.082) 0.0777 |
| **Q3** | 1,265 | 0.85 (0.613, 1.172) 0.3169 | 0.70 (0.445, 1.098) 0.1200 | 0.95 (0.586, 1.527) 0.8198 | 1.23 (0.576, 2.629) 0.5916 |
| **Q4** | 1,203 | 0.80 (0.571, 1.119) 0.1916 | 0.73 (0.436, 1.212) 0.2218 | 0.96 (0.583, 1.567) 0.8562 | 1.05 (0.440, 2.507) 0.9111 |
| **Q5** | 1,309 | 0.72 (0.493, 1.039) 0.0782 | **0.41 (0.221, 0.764) 0.0050** | 1.15 (0.685, 1.921) 0.6015 | 0.65 (0.252, 1.687) 0.3785 |
| **P for trend** | 6,275 | **0.0492** | **0.0067** | 0.6984 | 0.9289 |
| **>=50 years** |  |  |  |  |  |
| **Q1** | 1,249 | **1.0** | **1.0** | **1.0** | **1.0** |
| **Q2** | 1,323 | 0.72 (0.503, 1.037) 0.0779 | 0.84 (0.502, 1.419) 0.5218 | 0.89 (0.500, 1.570) 0.6783 | 1.06 (0.388, 2.906) 0.9076 |
| **Q3** | 1,291 | 0.71 (0.489, 1.025) 0.0675 | 0.80 (0.435, 1.477) 0.4782 | 1.22 (0.691, 2.147) 0.4949 | 1.36 (0.464, 3.994) 0.5751 |
| **Q4** | 1,334 | **0.63 (0.425, 0.934) 0.0215** | 0.61 (0.296, 1.254) 0.1788 | 0.89 (0.478, 1.638) 0.6976 | 1.18 (0.367, 3.802) 0.7791 |
| **Q5** | 1,238 | **0.44 (0.272, 0.694) 0.0005** | 0.68 (0.322, 1.449) 0.3210 | 1.02 (0.517, 2.016) 0.9535 | 0.95 (0.241, 3.735) 0.9390 |
| **P for trend** | 6,435 | **0.0013** | 0.1885 | 0.9387 | 0.9339 |

Adjust for: BMI (Smooth); C reactive protein (Smooth); Alcohol intake (Smooth); Fat intake (Smooth); Protein intake (Smooth); Total energy intake (Smooth); Family poverty income ratio (Smooth); Stroke; Gender; Hypertension history; Physical activity; Smoked at least 100 cigarettes in life.

**Supplementary Table 14.** Association between severe headache or migraine with fiber intake stratified by gender among different age groups (raw data).

| Stratified by gender | N | Age group (OR (95%CI) P-value) | |
| --- | --- | --- | --- |
|  |  | **<50 years** | **>=50 years** |
| Male |  |  |  |
| **Q1** | 1,076 | **1.0** | **1.0** |
| **Q2** | 1,038 | 0.79 (0.54, 1.15) 0.2180 | 1.09 (0.70, 1.70) 0.6959 |
| **Q3** | 1,217 | 0.91 (0.64, 1.31) 0.6107 | 1.12 (0.72, 1.75) 0.6180 |
| **Q4** | 1,370 | 0.91 (0.63, 1.32) 0.6184 | 0.85 (0.53, 1.36) 0.5064 |
| **Q5** | 1,664 | 0.80 (0.54, 1.18) 0.2676 | 0.72 (0.43, 1.21) 0.2195 |
| **P for trend** | 6,365 | 0.5705 | 0.1446 |
| Female |  |  |  |
| **Q1** | 1,460 | **1.0** | **1.0** |
| **Q2** | 1,496 | 0.94 (0.74, 1.20) 0.6306 | 0.70 (0.51, 0.95) 0.0221 |
| **Q3** | 1,339 | 0.82 (0.63, 1.07) 0.1424 | 0.75 (0.54, 1.03) 0.0780 |
| **Q4** | 1,167 | 0.76 (0.57, 1.02) 0.0702 | **0.66 (0.46, 0.95) 0.0262** |
| **Q5** | 8,83 | **0.67 (0.48, 0.94) 0.0218** | **0.55 (0.36, 0.84) 0.0062** |
| **P for trend** | 6,345 | **0.0096** | **0.0135** |

Adjust for: BMI (Smooth); C reactive protein (Smooth); Alcohol intake (Smooth); Fat intake (Smooth); Protein intake (Smooth); Total energy intake (Smooth); Family poverty income ratio (Smooth); Stroke; Hypertension history; Physical activity; Race; Smoked at least 100 cigarettes in life.

**Supplementary Table 15.** Association between severe headache or migraine with fiber intake quintiles stratified by different age (years) groups among females (raw data).

| **Stratification analysis** | **N** | | **Fiber intake quintiles (OR (95%CI) P-value)** | | | | | |
| --- | --- | --- | --- | --- | --- | --- | --- | --- |
|  |  |  | **Q1** | **Q2** | **Q3** | **Q4** | **Q5** | **P for trend** |
| **Age groups** |  | |  |  |  |  |  |  |
| <50 | 3,086 | | **1.0** | 0.94 (0.74, 1.20) 0.6306 | 0.82 (0.63, 1.07) 0.1424 | 0.76 (0.57, 1.02) 0.0702 | **0.67 (0.48, 0.94) 0.0218** | **0.0096** |
| >=50 | 3,259 | | **1.0** | **0.70 (0.51, 0.95) 0.0221** | 0.75 (0.54, 1.03) 0.0780 | **0.66 (0.46, 0.95) 0.0262** | **0.55 (0.36, 0.84) 0.0062** | **0.0135** |
| **Age groups** |  | |  |  |  |  |  |  |
| <40 | 1,953 | | **1.0** | 1.20 (0.88, 1.64) 0.2410 | 0.93 (0.67, 1.31) 0.6923 | 0.83 (0.57, 1.21) 0.3273 | 0.65 (0.42, 1.01) 0.0572 | **0.0159** |
| >=40, <60 | 1,990 | | **1.0** | **0.71 (0.52, 0.96) 0.0288** | 0.74 (0.52, 1.05) 0.099 | 0.70 (0.48, 1.01) 0.0595 | 0.69 (0.45, 1.05) 0.0855 | 0.1102 |
| >=60 | 2,402 | | **1.0** | 0.68 (0.45, 1.02) 0.0615 | 0.83 (0.55, 1.25) 0.3739 | 0.72 (0.45, 1.14) 0.1614 | **0.56 (0.32, 0.99) 0.0474** | 0.1275 |
|  |  |  | |  |  |  |  |  |

Adjust for: BMI (Smooth); C reactive protein (Smooth); Alcohol intake (Smooth); Fat intake (Smooth); Protein intake (Smooth); Total energy intake (Smooth); Family poverty income ratio (Smooth); Stroke; Hypertension history; Physical activity; Race; Smoked at least 100 cigarettes in life.

**Supplementary Table 16.** Association between severe headache or migraine with fiber intake quintiles stratified by different BMI (kg/m^2^) groups (raw data).

| **Stratification analysis** | **N** | **Fiber intake quintiles (OR (95%CI) P-value)** | | | | | |
| --- | --- | --- | --- | --- | --- | --- | --- |
|  |  | **Q1** | **Q2** | **Q3** | **Q4** | **Q5** | **P for trend** |
| BMI groups |  |  |  |  |  |  |  |
| <25 | 3,956 | **1.0** | 0.91 (0.68, 1.21) 0.5212 | 0.90 (0.67, 1.21) 0.4763 | **0.70 (0.50, 0.96) 0.0273** | **0.58 (0.40, 0.82) 0.0025** | **0.0014** |
| >=25, <30 | 4,446 | **1.0** | 0.97 (0.73, 1.28) 0.8072 | 0.90 (0.67, 1.22) 0.5155 | 0.96 (0.70, 1.32) 0.8221 | 1.00 (0.71, 1.42) 0.9871 | 0.9659 |
| >=30 | 3,956 | **1.0** | 0.83 (0.64, 1.07) 0.1525 | 0.94 (0.72, 1.23) 0.6512 | 0.84 (0.63, 1.13) 0.2431 | **0.64 (0.46, 0.90) 0.0110** | **0.0396** |
| BMI tertiles |  |  |  |  |  |  |  |
| Low (12.0-25.2) | 4,119 | **1.0** | 0.90 (0.68, 1.19) 0.4700 | 0.88 (0.66, 1.18) 0.3842 | **0.70 (0.51, 0.96) 0.0277** | **0.59 (0.42, 0.84) 0.0036** | **0.0014** |
| Middle (25.2-29.8) | 4,115 | **1.0** | 0.96 (0.72, 1.29) 0.7858 | 0.89 (0.65, 1.22) 0.4847 | 0.93 (0.67, 1.29) 0.6580 | 0.94 (0.66, 1.35) 0.7504 | 0.7388 |
| High (29.8-66.4) | 4,124 | **1.0** | 0.85 (0.66, 1.10) 0.2146 | 0.97 (0.75, 1.27) 0.8468 | 0.88 (0.66, 1.17) 0.3747 | **0.69 (0.49, 0.96) 0.0265** | 0.0830 |

Adjust for: Age (Smooth); C reactive protein (Smooth); Alcohol intake (Smooth); Fat intake (Smooth); Protein intake (Smooth); Total energy intake (Smooth); Family poverty income ratio (Smooth); Stroke; Gender; Hypertension history; Physical activity; Race; Smoked at least 100 cigarettes in life.

**Supplementary Table 17.** Demographics based on Headaches or migraines (raw data).

| Headaches or migraines | No  n=10,183 | Yes  n=2,527 | P-value* |
| --- | --- | --- | --- |
| Age, years | 52.6 ± 19.1 | 45.0 ± 16.2 | <0.001 |
| Family poverty income ratio | 2.7 ± 1.6 | 2.3 ± 1.5 | <0.001 |
| **C reactive protein, mg/dL** | 0.5 ± 1.0 | 0.5 ± 0.8 | <0.001 |
| **BMI, Kg/m^2^** | 28.2 ± 6.0 | 28.9 ± 6.9 | <0.001 |
| **Total energy intake, Kcal/d** | 2091.4 ± 1007.5 | 2064.9 ± 1053.8 | 0.049 |
| **Fat intake, g/d** | 77.6 ± 45.2 | 76.1 ± 47.6 | 0.014 |
| **Protein intake, g/d** | 79.3 ± 41.6 | 76.2 ± 44.4 | <0.001 |
| **Fiber intake, g/d** | 15.9 ± 10.1 | 14.5 ± 9.6 | <0.01 |
| **Alcohol intake, g/d** | 10.8 ± 33.1 | 7.1 ± 23.8 | <0.001 |
| **Gender** |  |  | <0.0001 |
| Male | 5485 (53.9%) | 880 (34.8%) |  |
| Female | 4698 (46.1%) | 1647 (65.2%) |  |
| **Race** |  |  | <0.001 |
| Non-Hispanic white | 5320 (52.2%) | 1158 (45.8%) |  |
| Non-Hispanic black | 1950 (19.1%) | 541 (21.4%) |  |
| Mexican American | 2218 (21.8%) | 614 (24.3%) |  |
| Other Race | 695 (6.8%) | 214 (8.5%) |  |
| **Stroke** |  |  | 0.028 |
| Yes | 358 (3.5%) | 112 (4.4%) |  |
| No | 9816 (96.5%) | 2410 (95.6%) |  |
| **Smoke** |  |  | 0.511 |
| Yes | 5048 (49.6%) | 1235 (48.9%) |  |
| No | 5121 (50.4%) | 1290 (51.1%) |  |
| **Hypertension history** |  |  | 0.617 |
| Yes | 3355 (33.3%) | 822 (32.8%) |  |
| No | 6712 (66.7%) | 1684 (67.2%) |  |
| **Physical activity** |  |  | 0.011 |
| sits | 2555 (25.1%) | 715 (28.3%) |  |
| stands or walks | 5396 (53.1%) | 1280 (50.7%) |  |
| light load | 1540 (15.1%) | 361 (14.3%) |  |
| Heavy work | 676 (6.6%) | 171 (6.8%) |  |
| **Fiber intake (g/d) quintiles** |  |  | <0.001 |
| Q1 (0.00-7.79) | 1928 (18.9%) | 608 (24.1%) |  |
| Q2 (7.80-11.59) | 2002 (19.7%) | 532 (21.1%) |  |
| Q3 (11.60-15.70) | 2057 (20.2%) | 499 (19.7%) |  |
| Q4 (15.71-22.08) | 2079 (20.4%) | 458 (18.1%) |  |
| Q5 (22.10-95.50) | 2117 (20.8%) | 430 (17.0%) |  |

*Kruskal−Wallis test

**Supplementary Table 18.** Univariate analysis of the association between severe headache or migraine with related variables (raw data).

|  | **N** | **Statistics** | **OR** | **95% CI** | **P-value** |
| --- | --- | --- | --- | --- | --- |
| **Gender** |  |  |  |  |  |
| Male | 6365 | 50.08% | 1.0 |  |  |
| Female | 6345 | 49.92% | 2.19 | 2.00, 2.39 | <0.0001 |
| **Race** |  |  |  |  |  |
| Non-Hispanic white | 6478 | 50.97% | 1.0 |  |  |
| Non-Hispanic black | 2491 | 19.60% | 1.27 | 1.14, 1.43 | <0.0001 |
| Mexican American | 2832 | 22.28% | 1.27 | 1.14, 1.42 | <0.0001 |
| Other race | 909 | 7.15% | 1.41 | 1.20, 1.67 | <0.0001 |
| **Stroke** |  |  |  |  |  |
| Yes | 470 | 3.70% | 1.0 |  |  |
| No | 12226 | 96.30% | 0.78 | 0.63, 0.97 | 0.0285 |
| **Smoked at least 100 cigarettes in life** |  |  |  |  |  |
| Yes | 6283 | 49.50% | 1.0 |  |  |
| No | 6411 | 50.50% | 1.03 | 0.94, 1.12 | 0.5113 |
| **Hypertension history** |  |  |  |  |  |
| Yes | 4177 | 33.22% | 1.0 |  |  |
| No | 8396 | 66.78% | 1.02 | 0.93, 1.12 | 0.6173 |
| **Physical activity** |  |  |  |  |  |
| sits | 3270 | 25.76% | 1.0 |  |  |
| stands or walks | 6676 | 52.59% | 0.85 | 0.76, 0.94 | 0.0016 |
| light load | 1901 | 14.98% | 0.84 | 0.73, 0.96 | 0.0141 |
| heavy work | 847 | 6.67% | 0.90 | 0.75, 1.09 | 0.2901 |
| **Age** | 12,710 | 51.06 ± 18.80 | 0.98 | 0.98, 0.98 | <0.0001 |
| **Family poverty income** | 11,610 | 2.61 ± 1.60 | 0.85 | 0.82, 0.87 | <0.0001 |
| **C reactive protein, mg/dl** | 12,075 | 0.48 ± 0.96 | 1.04 | 0.99, 1.08 | 0.1035 |
| **BMI** | 12,358 | 28.33 ± 6.24 | 1.02 | 1.01, 1.03 | <0.0001 |
| **Energy intake, kcal/day** | 12,710 | 2086.12 ± 1016.86 | 1.00 | 1.00, 1.00 | 0.2404 |
| **Fat intake, g/day** | 12,710 | 77.31 ± 45.71 | 1.00 | 1.00, 1.00 | 0.1528 |
| **Protein intake, g/day** | 12,710 | 78.68 ± 42.23 | 1.00 | 1.00, 1.00 | 0.0012 |
| **Alcohol intake, g/day** | 12,710 | 10.09 ± 31.55 | 0.99 | 0.99, 1.00 | <0.0001 |

**
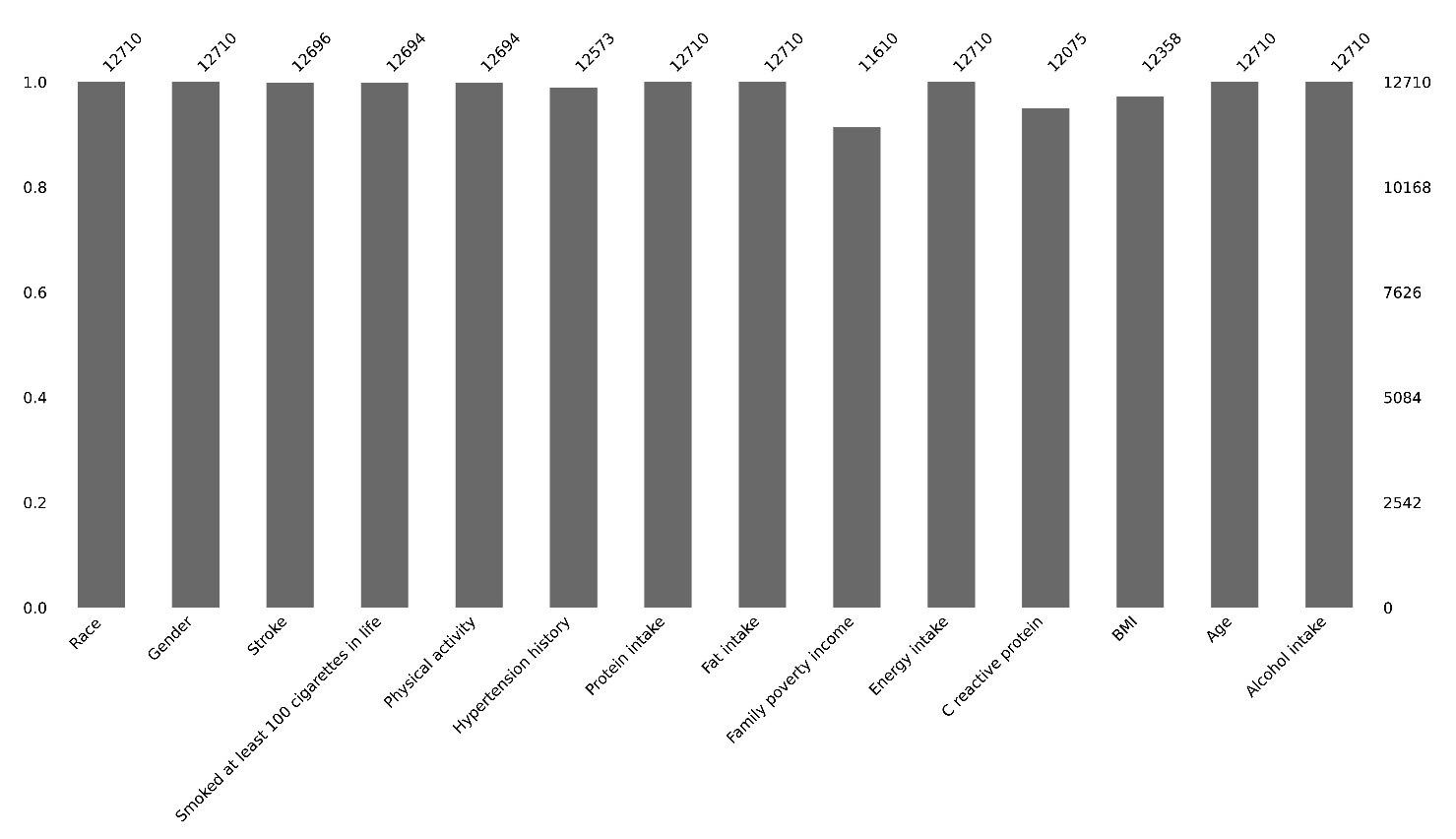
**

**Supplementary Figure 1.** The non-missing data of all covariates (raw data). The gray bars represent the number of per-variable non-missing values. This figure was produced with Python (the package of missingno, Python version 3.7.2).
